# Successful Treatment of Facial Merkel Cell Carcinoma Using CyberKnife Radiotherapy Alone: A Case Report

**DOI:** 10.7759/cureus.50699

**Published:** 2023-12-17

**Authors:** Shinichiro Mizumatsu, Toshiharu Fujiyama

**Affiliations:** 1 CyberKnife Center, Aoyama General Hospital, Toyokawa, JPN; 2 Department of Dermatology, Hamamatsu University School of Medicine, Hamamatsu, JPN

**Keywords:** facial merkel cell carcinoma, stereotactic body radiotherapy (sbrt), stereotactic radiotherapy (srt), cyberknife radiotherapy, radiotherapy alone, facial skin cancer, elder, radiotherapy, cyberknife, merkel cell carcinoma

## Abstract

Merkel cell carcinoma (MCC) is a rare and aggressive neuroendocrine skin tumor. Herein, we present a case of MCC which was successfully treated with radiotherapy alone using CyberKnife® (CK) (Accuray Incorporated, Sunnyvale, California, United States). An 86-year-old female patient presented with multiple painless pink rash skin tumors on the left cheek. The patient was diagnosed with MCC based on a lesion biopsy (T2cN2M0, stage IIIB). The patient was referred for CK radiotherapy (CKR) at our institution because of her advanced age and inoperative lesions. The patient underwent CKR alone, with a planning target volume (PTV) of 14.9 ml, a prescribed dose of 30 Gy, a maximum dose of 46.2 Gy, and an isodose line (the minimum dose of 95% of the PTV) of 65% in 10 fractions for 13 days. The lesions had completely regressed on the last day of CKR. Left cervical lymph node metastasis (CLNM) appeared 10 months after CKR. The patient underwent a second CKR for CLNM, the PTV was 4.6 ml, and the prescribed dose was 27 Gy in three fractions for three days. The CLNM had completely regressed one month later after the second CKR. Primary lesions did not recur for 33 months after the initial CKR, and CLNM did not reappear for 23 months after the second CKR with good cosmetic results. No CKR-related adverse event occurred in our follow-up period. Our present case indicates that CKR is an effective treatment option for patients with MCC, particularly elderly patients who may not be suitable candidates for extensive surgical resection.

## Introduction

Merkel cell carcinoma (MCC), a rare and aggressive neuroendocrine skin tumor with increasing incidence and mucous membrane, was first described by Toner in 1972 [1]. It most frequently presents on the head and neck region of the elderly [2-3]. The diagnosis requires histological validation, using immunohistochemistry, including cytokeratin-20 (CK-20) and thyroid transcription factor-1 (TTF-1) immunomarkers, to avoid misdiagnosis with the lymphoma or small-cell lung carcinoma metastasis [2]. The standard treatment for MCC includes extensive surgical resection and postoperative radiotherapy. There is a systematic review that three-year local control was 20% (median 10%) in the observation cohort, compared to 65% (62%) with postoperative radiotherapy (RT) and 67% (75%) with postoperative chemotherapy plus radiation therapy (chemoRT) in the postoperative 4475 patients [4]. Surgery is not always possible due to the localization or comorbidities of elderly patients. Radiotherapy is a safe and non-invasive alternative for patients who do not undergo surgery because MCC is highly radiosensitive [5]. Herein, we report a case of an elderly patient with facial MCC who was successfully treated with CyberKnife® (CK) (Accuray Incorporated, Sunnyvale, California, United States) alone [6].

## Case presentation

**Figure 1:**
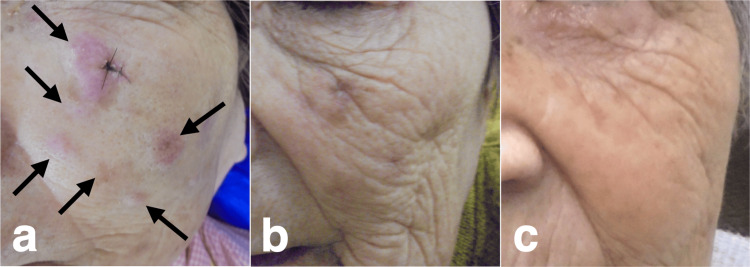
Time course of clinical findings on photography. (a) Multiple pink-colored nodules on the left cheek skin before CKR (arrows). (b) The lesions had completely regressed, leaving marks on the last day of CKR (13 days after CKR started). (c) Absence of recurrence lesion on the left cheek skin 30 months after CKR. CKR: CyberKnife® (Accuray Incorporated, Sunnyvale, California, United States) radiotherapy.

The patient was an 86-year-old female. The patient's medical history included a neck clipping of the left middle cerebral artery aneurysm, rheumatoid arthritis, and age-related dementia. The patient presented with multiple painless nodules on the left cheek skin two months ago. A biopsy confirmed MCC. The patient was referred for CK radiotherapy (CKR) at our institution because of her advanced age and inoperative lesions. When the patient first visited our hospital, multiple pink-colored nodules were scattered on the left cheek skin (Figure 1a).

**Figure 2:**
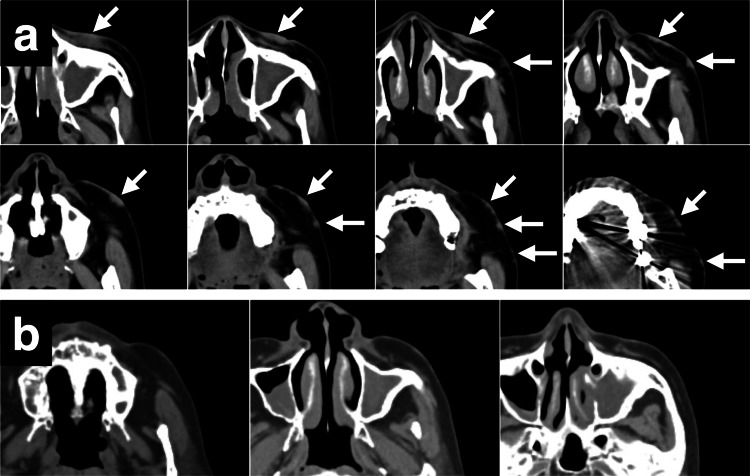
Axial CT of the left cheek before and after CKR. (a) CT showing multiple lesions on the surface of left cheek skin before CKR (arrows). (b) CT showing the disappearance of lesions the next day after completing CKR (14 days after CKR started). CKR: CyberKnife® (Accuray Incorporated, Sunnyvale, California, United States) radiotherapy.

CT revealed multiple lesions that spread thinly and dotted on the skin surfaces, with no metastatic lesion in the body (Figure 2a, 3a). The maximum lesion size was 2.5 cm with in-transit metastases. TNM (tumor (T), extent of spread to the lymph nodes (N), and presence of metastasis (M)) classification was T2cN2M0, and the clinical stage was IIIB (The American Joint Committee on Cancer, TNM staging 8th edition, 2017).

**Figure 3:**
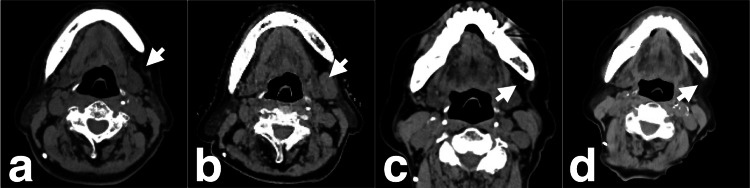
Axial CT of a left cervical lymph node metastasis before and after CKR. (a) CT showing no lesion before the initial CKR (arrow); (b) CT showing a lesion before the second CKR (arrow); (c) CT showing the disappearance of the lesion one month after the second CKR (arrow); (d) CT showing no recurrence 20 months after the second CKR (arrow). CKR: CyberKnife® (Accuray Incorporated, Sunnyvale, California, United States) radiotherapy.

**Figure 4:**
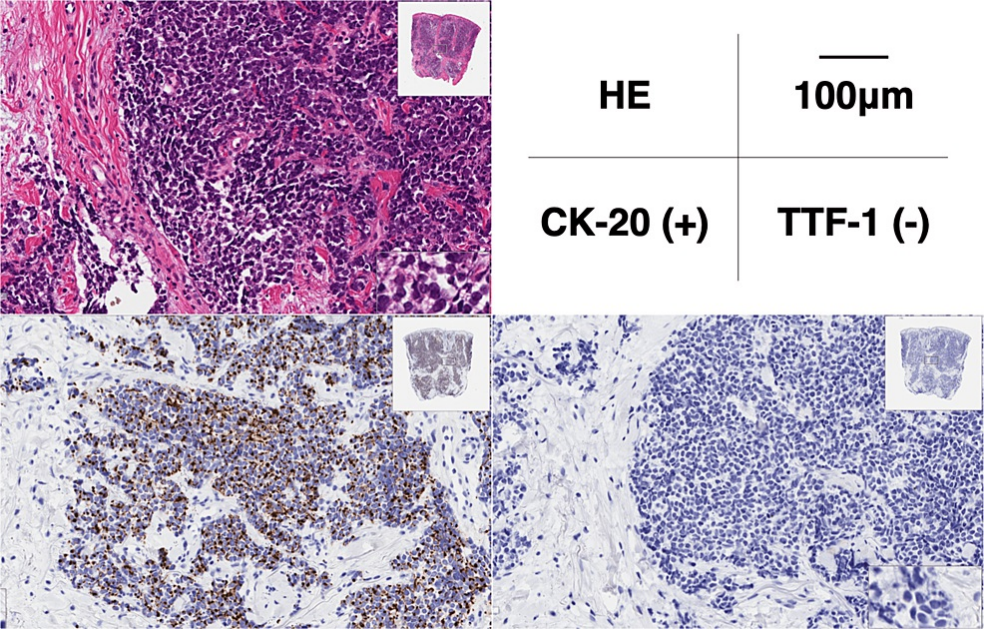
Histopathological findings. The specimen included the left cheek skin biopsy tissue. H&E staining revealed solid alveolar nest-like proliferation of atypical cells with a high N/C ratio in the dermis. Immunohistochemical staining was positive for CK-20 and negative for TTF-1. N/C: nuclear/cytoplasmic, CK-20: cytokeratin-20, TTF-1: thyroid transcription factor-1.

We performed a biopsy of skin tissue on the patient's left cheek. Histopathological examination was as follows (Figure 4): H&E staining revealed solid alveolar nest-like proliferation of atypical cells with a high nuclear/cytoplasmic (N/C) ratio in the dermis. Immunohistochemical staining was positive for CK-20, synaptophysin, and CD56 and negative for TTF-1, CK-7, CD3, and chromogranin A. Histopathological diagnosis was consistent with MCC.

**Figure 5:**
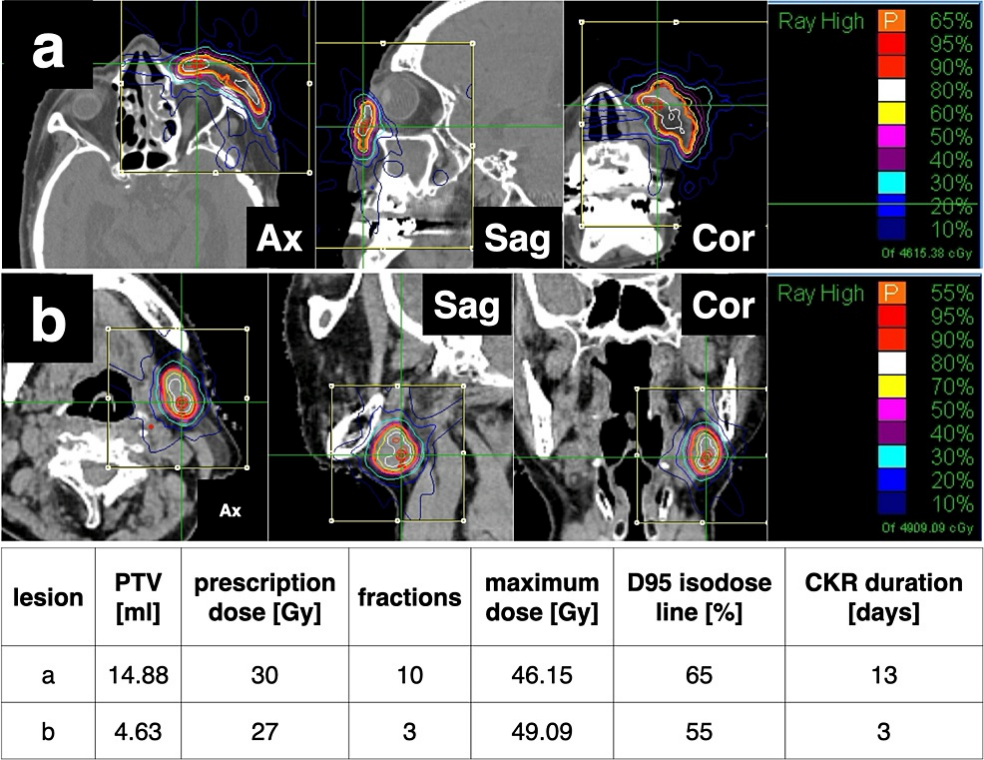
Dose distribution of CKR plans. (a) The plan of primary lesions on the left cheek skin. (b) The plan of the left cervical lymph node metastasis. CKR: CyberKnife® (Accuray Incorporated, Sunnyvale, California, United States) radiotherapy.

We created a CKR plan using MultiPlan® (Accuray Incorporated, Sunnyvale, California, United States) (Figure 5a).

The contouring of the tumor and critical organs was defined with CT imaging. The lesions were contoured as a single mass because accurately determining the gross tumor volume (GTV) was difficult for each individual. The planning target volume (PTV) was obtained by the GTV plus an expansion of 2-5 mm toward the dermis and reduced by 1 mm from the skin surface. The CKR planning was obtained with an inverse planning algorithm using a nonisocentric technique. Six dimensional-skull tracking was used as the tracking method. We utilized a fixed collimator with 10.0 mm. We did not use a beam passing through the eyeball. The volume of the PTV was 14.9 ml. The dose to the target was 30 Gy delivered in 10 fractions in 13 days, prescribed to the 65% isodose line. The maximum dose (Dmax) was 46.2 Gy. The coverage of the target was 95%, the number of beams was 164, the conformity index was 1.25, the new conformity index was 1.32, and the homogeneity index was 1.54. The total monitor unit was 51039. The Dmax at the critical organs for all 10 fractions were 6.9 Gy, 5.1 Gy, 1.3 Gy, 3.0 Gy, and 38.3 Gy at the left eye, left optic nerve, right eye, right optic nerve, and left cheek skin surface, respectively. The patient underwent CKR in 10 fractions for 13 days as planned. The lesions had completely regressed leaving marks on the last day of CKR, and with no adverse event during the CKR period (Figure 1b, 2b).

A left cervical lymph node metastasis (CLNM) appeared during the follow-up after initial CKR (Figure 3b).

The patient underwent a second CKR for CLNM 10 months after CKR, with a PTV of 4.6 ml, a prescribed dose of 27 Gy, and a Dmax of 49.1 Gy in three fractions for three days (Figure 5b). The CLNM had completely regressed one month after the second CKR (Figure 3c). Primary lesions did not recur for 33 months after the initial CKR, and CLNM did not reappear for 23 months after the second CKR with good cosmetic results (Figure 1c, 3d). CKR-related adverse events and worsening of her symptoms did not occur in our follow-up period.

## Discussion

MCC is a rare disease that accounts for 0.6% of all skin cancers in Japan, and the mean age of 80.7 years was the highest, indicating that it was more likely to occur in the elderly [3]. The standard treatment for MCC is wide excision with or without radiotherapy depending on surgical findings [2]. However, general anesthesia and surgery are associated with high risks for elderly patients. Further, wide excision is difficult in the head and neck region due to functional and cosmetic problems. Hence, radiotherapy may be a radical treatment alternative to surgery because MCC demonstrated high radiosensitivity. Patel et al. [7] reported that radiation monotherapy demonstrated comparable results to MCC standard therapy in a review. Most radiation monotherapy reports included conventional radiotherapy (CRT) and electron beam therapy (EBT) [7-11]. However, CRT causes severe side effects and significantly reduces the quality of life, especially when used near critical structures where standard wide-margin surgical therapies may be impractical. 

Stereotactic body radiotherapy (SBRT) is a cancer treatment that delivers extremely precise, very intense radiation doses to cancer cells while minimizing damage to healthy tissue. SBRT is more advantageous than CRT because it precisely irradiates tumors while allowing a tight margin of surrounding normal tissues. EBT is frequently used for skin tumors, but SBRT is superior in terms of high irradiation and dose distribution accuracies [12,13]. CK is an SBRT device with image guidance, and it consists of a robot arm, linear accelerator, and target tracking system. This system irradiates a target with less damage to proximal organs by moving the robot arm, which has a wide range of motion. CKR is less invasive compared with standard surgery and demonstrates a shorter treatment period and higher efficacy than CRT [13-15]. 

Two studies reported CKR for MCC, to the best of our knowledge [14,15]. Tuskada et al. [14] reported that CKR for eyelid MCC successfully resulted in irradiation doses ranging from 50 Gy in five fractions. The tumor mass had completely regressed to a scar one month after CKR, with no sign of local recurrence for six months. This report demonstrated no CKR-related severe side effects. Shirai et al. [15] reported a successful CKR for recurrent eyelid MCC after wide local excision and skin grafting. The tumor mass with an additional 3 mm of margin was irradiated with doses ranging from 35 Gy in five fractions by CK. This report demonstrated no local recurrence for two years after CKR. Zhu et al. [16] reported that EBT (8 Gy single irradiation) and SBRT (32 Gy in four fractions for two weeks) for MCC resulted in no residual tumor four weeks after treatment without adverse events although the device was not described. This report demonstrated no local recurrence for three months after EBT and SBRT. Pontoriero et al. [13] reported squamous cell facial carcinoma treated with CK. They used a fixed collimator with different diameters of 7.5, 10.0, and 12.5 mm. The dose to the target was 42 Gy delivered in six sessions in three weeks (two fractions per week) prescribed to the 80% isodose line. The Dmax was 52.5 Gy and the coverage of the target was 96.7%. CKR completely regressed the tumor after 12 months with no recurrence for 24 months. However, the patient demonstrated an acute reaction characterized by desquamation, dermatitis, ulceration, and skin damage that required treatment. These adverse events have since recovered.

The tolerable dose to skin with cancer invasion in SBRT remains currently unclear. Zaorsky et al. [17] reported good cosmesis in 80% of patients with skin cancer after radiotherapy under a biologically effective dose (BED) of 100 Gy (α/β = 3) in a meta-analysis. This BED dose corresponds to 50 Gy in 15 fractions, 36.7 Gy in seven fractions, and 35 Gy in five fractions as a treatment radiation dose. Protocols that extend the treatment duration, such as irradiation every alternate day, resulted in the reduction of adverse events, such as rectal toxicity in prostate cancer, carotid blowout syndrome in recurrent head and neck cancer, and general fatigue in hepatocellular carcinoma, after SBRT treatment [18-20].

Our experience demonstrated the efficacy of CKR in treating facial MCC. Since CKR is localized radiotherapy, it is necessary to carefully follow the patient after the treatment. However, more data are necessary to confirm our observation.

## Conclusions

We described a case of facial MCC that was successfully treated with CKR alone. There has been no recurrence of the primary lesion for 33 months and lymph node metastasis for 23 months after CKR alone. In addition, no adverse events or worsening of her symptoms occurred after CKR during our follow-up. CKR may be an effective local treatment for MCC in the elderly and patients with inoperable tumors. However, the details of CKR for MCC, such as the optimal dose, number of fractions, and treatment period, have not been determined. It is necessary to accumulate additional clinical data on whether CKR is an effective treatment option for MCC.
